# Metastatic Squamous Cell Carcinoma in the Gallbladder Fossa Complicated by a Duodenal Fistula

**DOI:** 10.7759/cureus.6522

**Published:** 2019-12-31

**Authors:** Thomas P Reith, Vaisak Nair, Edward J McKenna, Arun Singavi

**Affiliations:** 1 Internal Medicine, Medical College of Wisconsin, Milwaukee, USA; 2 Hematology and Oncology, Medical College of Wisconsin, Milwaukee, USA

**Keywords:** squamous cell carcinoma, cancer of unknown primary, squamous cell carcinoma of unknown primary, duodenal fistula, gallbladder fossa

## Abstract

Cancer of unknown primary is defined as a metastatic disease present in the absence of an identifiable primary site of origin. Squamous cell carcinoma (SCC) of unknown primary is a relatively uncommon subtype that usually involves metastases to the cervical or inguinal lymph nodes. We present a rare case of SCC of unknown primary metastasizing to the gallbladder fossa and creating a duodenal fistula. This case highlights the rarity of SCCs in the gallbladder region and the risks posed by chemotherapy in patients with gastrointestinal fistulas.

## Introduction

Cancer of unknown primary (CUP) is defined as a metastatic disease present in the absence of an identifiable primary site of origin despite a thorough clinical examination and diagnostic workup [1]. Accounting for approximately 3-5% of all cancers, CUP has a poorly understood natural history and confers a poor prognosis [2]. CUP is subclassified into the following four categories based on histological examination of the initial biopsy: adenocarcinomas, squamous cell carcinomas (SCCs), neuroendocrine carcinomas, and poorly or undifferentiated tumors [1]. SCC of unknown primary is relatively uncommon and usually involves primary cancers of the head and neck, lung, or anogenital region metastasizing to the upper cervical, lower cervical, or inguinal lymph nodes, respectively. Metastatic SCC presenting at other sites is very rare, and empiric chemotherapy is the standard treatment. Here, we present a case of SCC of unknown primary metastasizing to the gallbladder fossa and creating a duodenal fistula.

## Case presentation

**Figure 1:**
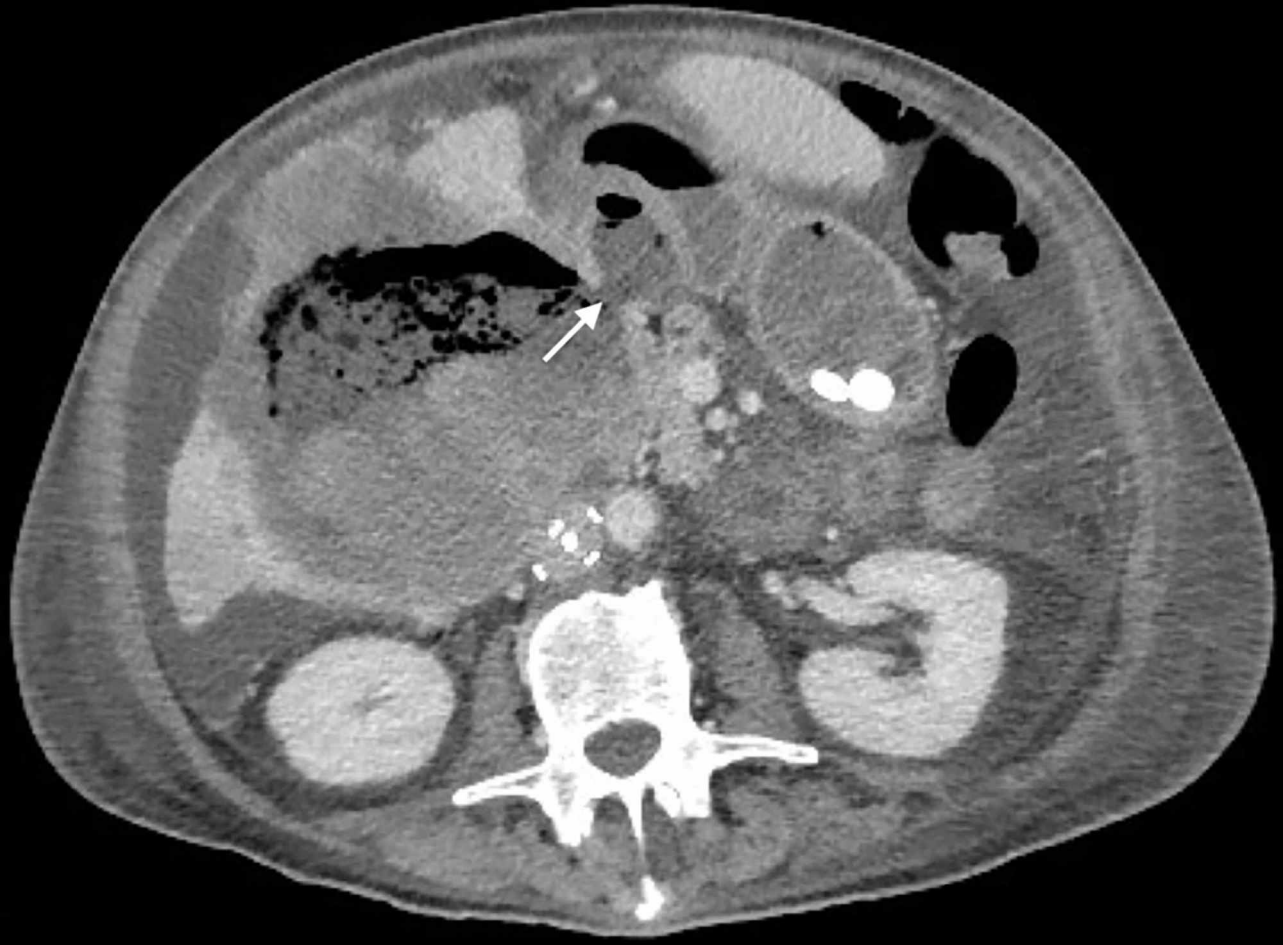
Coronal view of abdominal CT showing a large mass involving the central liver. The arrow shows the mass fistulizing to the duodenum.

A 68-year-old man presented to the emergency department with right upper quadrant pain, lower extremity swelling, and weight loss. Computed tomography (CT) showed a large necrotic mass in the gallbladder fossa fistulizing to the duodenum (Figure 1). A positron emission tomography (PET) scan revealed hypermetabolic liver metastases and portacaval lymphadenopathy, and an ultrasound-guided biopsy of the mass revealed keratinizing squamous cells. Subsequent upper endoscopy, chest CT, and anal exam failed to identify a primary lesion, and the patient was diagnosed with stage 4 metastatic SCC of unknown primary origin. Due to the metastases, the tumor was deemed unresectable. Because of the duodenal fistula, it was feared that any myelosuppression induced by chemotherapy would pose a high risk of bacteremia. Consequently, chemotherapy was not administered initially.

**Figure 2:**
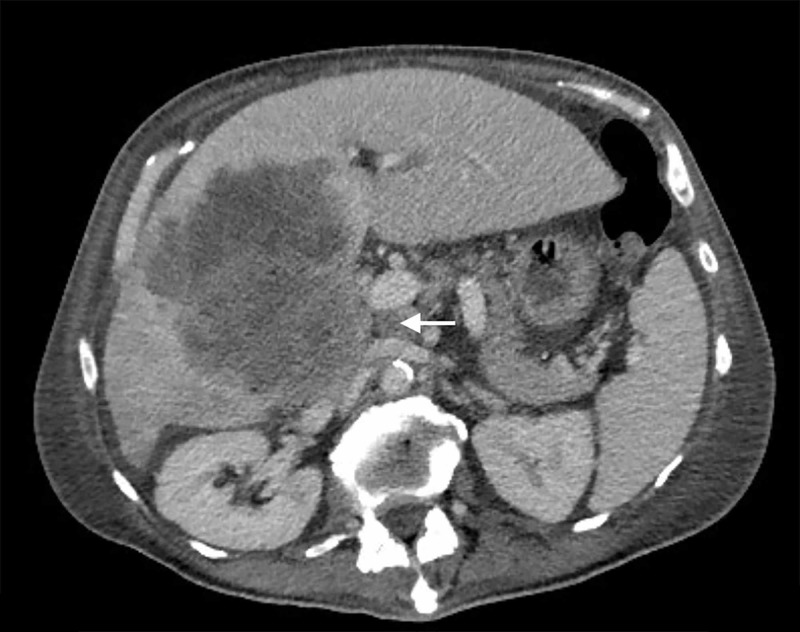
Coronal view of abdominal CT showing the mass compressing the suprarenal inferior vena cava at the point indicated by the arrow.

Over the next week, the patient’s edema progressively worsened to include the scrotum and abdomen, with both legs weeping clear fluid. A second abdominal CT revealed these symptoms to be secondary to the mass, which was compressing the suprarenal inferior vena cava (IVC) (Figure 2). At this point, the palliative benefits of chemotherapy were thought to outweigh the risks, and the patient was treated with low-dose carboplatin and paclitaxel. Additionally, he underwent targeted radiation therapy in an attempt to relieve the mass effect symptoms. Nevertheless, the disease progressed and the patient ultimately enrolled in hospice care and died two months later.

## Discussion

Differential diagnoses for a mass in the gallbladder fossa include gallbladder carcinoma, cholangiocarcinoma, and, less likely, metastatic disease. Primary biliary tract cancers normally arise in the setting of chronic inflammation, which promotes mucosal dysplasia and eventual malignancy. Accordingly, the largest risk factor for gallbladder carcinoma is cholelithiasis, with more than 70% of cases involving a history of gallstones [3]. On imaging, a mass replacing the gallbladder is the most common presentation [3]. The largest risk factor for cholangiocarcinoma in the Western populations is primary sclerosing cholangitis; in East Asia, infection by the liver flukes Clonorchis sinensis and Opisthorchis viverrini pose additional risks [4].

Although the vast majority of biliary tract cancers are adenocarcinomas [5], primary SCCs of structures in this region are nevertheless possible. Pure SCCs of the gallbladder account for 2% of all gallbladder malignancies [5]; SCCs originating from the biliary tree and the ampulla of Vater are even rarer, with around 25 cases of the former [6] and 6 of the latter [7,8] reported in the literature. Primary SCCs of the liver are also possible, with around 35 cases reported worldwide [9]. In this case, given the tumor’s size, location, and aggressiveness, as well as the absence of cervical and inguinal lymph node involvement, SCC originating from a hepatobiliary structure is plausible.

While the exact pathogenesis of hepatobiliary SCC is unknown, it is assumed that chronic inflammation of the biliary epithelium may promote squamous metaplasia and subsequent dysplasia and malignant transformation [10]. Predisposing conditions to hepatobiliary SCCs may thus be primary sclerosing cholangitis, parasitic liver fluke infection, and recurrent cholangitis. A second theory is the presence of heterotropic squamous epithelium, which may explain cases with no history of chronic inflammation [6]. Regardless of their etiology, primary gallbladder, biliary, and hepatic SCCs present at an advanced stage and have a poor prognosis [10-12].

A significant complication of this case was the duodenal fistula, which potentially allowed intestinal bacteria to enter the bloodstream. Since a common side effect of chemotherapy is myelosuppression, we initially held its administration since we feared complications of bacteremia and septic shock. After the mass compressed the IVC, however, we felt that the palliative benefit of chemotherapy outweighed any risk of sepsis. This case thus highlights that the risks and benefits of chemotherapy must be carefully considered in patients with gastrointestinal fistulas.

## Conclusions

SCCs in the gallbladder region are rare malignancies that present at an advanced stage and have a poor prognosis. Physicians should carefully consider the risks of chemotherapy in patients with gastrointestinal fistulas, as myelosuppression increases the risk of bacteremia. Moreover, physicians should keep in mind that malignancies of the gallbladder and biliary tree may obstruct the IVC.
